# Management of patients with seasonal allergic rhinitis: Diagnostic consideration of sensitization to non‐frequent pollen allergens

**DOI:** 10.1002/clt2.12058

**Published:** 2021-10-04

**Authors:** Conny Höflich, Galina Balakirski, Zuzanna Hajdu, Jens Malte Baron, Katharina Fietkau, Hans F. Merk, Ulrich Strassen, Henning Bier, Wolfgang Dott, Hans‐Guido Mücke, Wolfgang Straff, Gerda Wurpts, Amir S. Yazdi, Adam Chaker, Stefani T. M. Röseler

**Affiliations:** ^1^ German Environment Agency Section II 1.5 Environmental Medicine and Health Effects Assessment Berlin Germany; ^2^ Department of Dermatology and Allergology University Hospital RWTH Aachen Aachen Germany; ^3^ Department of Otorhinolaryngology Klinikum rechts der Isar Technical University Munich Munich Germany; ^4^ Department for Environmental Medicine University Hospital RWTH Aachen Aachen Germany; ^5^ Center of Allergy and Environment (ZAUM) Technical University and Helmholtz Center Munich Munich Germany; ^6^ Present address: Department of Dermatology, Allergology and Dermatosurgery HELIOS University Hospital Wuppertal University of Witten/Herdecke Wuppertal Germany; ^7^ Present address: Department of Pneumology, Allergology, Sleep and Respiratory Medicine Augustinians Hospital Cologne Germany

**Keywords:** allergic rhinitis, allergy, monitoring, pollen, sensitization

## Abstract

**Background:**

Diagnosis of pollen allergies is mainly based on test allergens for skin prick testing. In the minimum battery of test inhalant allergens recommended by the Global Allergy and Asthma European Network 10 pollen allergens are included. Complementary other pollen allergens may need to be considered; however, respective awareness may not always be granted. Furthermore, at least in Germany, the situation may be even more complicated by the fact that test allergens need regulatory approval. A decline in commercially available test allergens may result in a diagnostic gap regarding patients with non‐frequent allergies. How many patients with non‐frequent pollen allergies would be affected by this gap? The data presented here partly answer this question.

**Methods:**

The study consisted of a descriptive and an analytical part. In the descriptive part, sensitization to frequent pollen allergens (alder, hazel, birch, sweet grasses; according to the German Therapy Allergen Ordinance) and to respective non‐frequent pollen allergens (cypress, Japanese cedar, ash, plane tree, olive, Bermuda grass, wall pellitory, plantain, goosefoot, mugwort, ragweed, and saltwort) was measured in adult patients with physician‐diagnosed allergic rhinitis from two German federal states, namely North‐Rhine Westphalia (*n* = 360) and Bavaria (*n* = 339), using skin prick testing and/or ISAC technology. Furthermore, respective regional pollen data were assessed. In the analytical part, sensitization data were correlated with each other and with anamnestic data on symptom periods.

**Results:**

Sensitization to frequent pollen allergens ranged from 45% (sIgE to Aln g 1/Alder, NRW) to 72% (prick test reactivity to birch, NRW). Sensitization to non‐frequent pollen allergens ranged from 0% (sIgE to Amb a 1/ragweed, NRW) to 41% (prick test reactivity to olive, Bavaria). Sensitization data partly correlated with each other and in connection with symptom periods showed a partly similar seasonal pattern as pollen data.

**Conclusions:**

Sensitization to non‐frequent pollen allergens have to be considered when examining patients with respective seasonal symptoms, and test (and respective therapy) allergens for non‐frequent pollen allergies need to be available. Further prerequisites for adequate patient management would be a nationwide pollen monitoring system giving continuous pollen data and a systematic sensitization monitoring at patient level.

## 1 BACKGROUND

Allergens from pollen^1^ are the main cause of allergic respiratory diseases. In Germany, 15% of adults and 11% of children and adolescents suffer from allergic rhinitis, and concerning asthma, 9% and 5%, respectively, are affected (data from 2008 to 2011 [adults] and 2003 to 2006 [children and adolescents]).^1^, ^2^ In addition to respiratory symptoms, pollen allergens can induce oral symptoms due to cross‐reactivities to food allergens (so‐called Oral Allergy Syndrome or pollen‐related food allergy syndrome)^3^ and, although rather rarely, anaphylaxis.^4^

At present, pollen from sweet grasses (*Poaceae*; including Timothy grass, excluding Bermuda grass) and the birch family (*Betulaceae*; including among others birch, alder and hazel) are the most common clinically relevant pollen types in Germany: Almost every fifth of the German adult population is sensitized to Timothy grass pollen and birch pollen, respectively,^5^ and in adult patients with suspected allergic reaction to inhalant allergens and sensitization to sweet grasses or birch pollen, this sensitization was clinically relevant in 90% and 91%, respectively.^6^

Diagnosis of pollen allergies is mainly based on test allergens for skin prick testing. In 2009, the Global Allergy and Asthma European Network (GA^2^LEN) has recommended a standardized test allergen battery for clinical use and research, which allowed the identification of the majority of sensitized subjects studied.^6^, ^7^, ^8^ With respect to pollen, this battery included alder (*Alnus*), birch (*Betula*), cypress (*Cupressus*), hazel (*Corylus*), mugwort (*Artemisia*), olive (*Olea europaea*), plane tree (*Platanus*), ragweed (*Ambrosia*), sweet grasses (*Poaceae*; except corn), and wall pellitory (*Parietaria judaica*).^8^ Complementary, occasionally other allergens beyond that pollen panel may need to be considered when examining patients with seasonal allergic symptoms, for example, goosefoot or plantain pollen, but not always respective awareness can be assumed.

In Germany, the situation may be even more difficult because of the fact that test allergens are considered medicinal products and thus need regulatory approval by the Paul‐Ehrlich‐Institut, the Federal Institute for Vaccines and Biomedicines, thereby ensuring its quality, efficacy and safety. This means in effect, that phase I, II and III clinical studies have to be conducted prior to their marketing authorization.^9^ On the part of pharmaceutical companies, this might result in an uneconomic cost‐benefit‐ratio, when it comes to rare allergens, and thus, the approval of test allergens for rare allergies are possibly being pushed aside. Indeed, in the past years, a marked decrease in the number of approved test allergens has occurred in Germany.^9^

Notwithstanding the above‐described difficulties, patients with allergies to rare allergens have the same right for adequate diagnosis and therapy as patients with allergies to frequent allergens.^9^, ^10^ In addition, a social aspect has to be considered: Allergens rare today maybe frequent tomorrow as shown for instance for ragweed pollen and respective sensitization in Italy.^11^ Thus, monitoring sensitization to rare allergens would indicate changes in the allergen spectrum as expected for instance in the course of climate change.^12^

According to the European Union (EU) a disease is defined as rare if not more than 5 out of 10,000 people in the EU are affected by it.^10^ This definition could also be used to define rare allergies, and indeed, the German Therapy Allergen Ordinance (TAO), in effect since 2008, seems to have used this definition.^10^, ^13^ According to the TAO, currently allergens of the following sources can be considered as frequent allergens in Germany (botanical names in brackets): pollen of alder (*Alnus*), pollen of hazel (*Corylus*), pollen of birch (*Betula*), pollen of sweet grasses (*Poaceae*; except corn), dust mite (*Dermatophagoides*), bee venom and wasp venom. All other allergens including other pollen allergens and occupational allergens, for instance, would be considered as rare or—as we do here—non‐frequent allergens.

The decline in the number and the spectrum of commercially available test allergens may result in a diagnostic (and respective therapeutic) gap regarding patients with non‐frequent allergies.^9^, ^10^, ^14^ With respect to pollen, this would include allergies to mugwort or ragweed, but also allergies to ash, goosefoot, or plantain, among others.

How many people may be affected by this diagnostic gap? The above cited GA^2^LEN skin test study I included patient data on 10 pollen allergens, among them six non‐frequent allergens, from two German cities.^7^ On the basis of patient data from two German federal states, here, we expand the list of tested allergens to another six non‐frequent allergens using skin prick testing and/or sIgE analysis and complete sensitization data by anamnestic data on complaint periods and regional pollen data.

## 2 METHODS

### 2.1 Patients

The present analysis was based on patient data collected in the context of a study on climate change and allergy funded by the German Federal Ministry for the Environment, Nature Conservation, and Nuclear Safety (Ufoplan research grant number FKZ 3710 61 228).^15^ In this study, 952 patients with suspected allergic respiratory diseases had been recruited between May 2011 and July 2013: 476 from the German federal state North‐Rhine Westphalia (NRW), located in the western part of the country, and 476 from the German federal state Bavaria, located in the south part. In NRW, the study had been conducted at the Department of Dermatology and Allergology of the University Hospital of the Rhineland‐Westphalian Technical College Aachen. In Bavaria, the Department of Otorhinolaryngology, Klinikum rechts der Isar, Technical University Munich, had been responsible. The study had been approved by institutional review boards of both participating centers. Patients had filled in a questionnaire, had participated in a medical interview, and had undergone skin prick testing as well as blood withdrawal for analysis of antigen‐specific IgE (sIgE) levels. For further study details see.^15^

For the present analysis, only patients with physician‐diagnosed allergic rhinitis and valid skin prick testing (criteria see below) were included (*n* = 699; NRW: *n* = 360, minimum/median/maximum age 20/39/65 years, female 65%; Bavaria: *n* = 339, minimum/median/maximum age 20/45/65 years, female 63%). Patients were attributed with “physician‐diagnosed allergic rhinitis,” if they had answered both of the consecutive questions affirmatively: (i) “Did you suffer or do you suffer from hay fever or allergic rhinitis?,” (ii) “Has the hay fever or allergic rhinitis ever been diagnosed or confirmed by a doctor?” Questions, given in German, were adopted from a standardized physician‐administered computer‐assisted personal interview (CAPI) used in the German health interview and examination survey for adults (DEGS1).^16^

### 2.2 Sensitization data

Sensitization data were analyzed by skin prick testing and/or measurement of sIgE in serum using ISAC technology.

Skin prick testing was performed according to the GA^2^LEN guidelines on harmonization of skin prick testing in Europe.^17^ Histamine dihydrochloride (10 mg/ml; ALK‐Abelló) was used as positive control, diluent (ALK‐Abelló) as negative control. Results were assessed after 15 min. Valid negative and positive controls provided (i.e. largest diameter of the negative control <2 mm and largest diameter of the positive control ≥3 mm, respectively), skin prick testing to an allergen extract was evaluated positive if the largest diameter of the wheal was ≥3 mm. Pollen allergen extracts to which prick test reactions were assessed are given in Table 1. In addition, dust mite allergen was included as perennial control allergen.

**TABLE 1:** Pollen allergen extracts used for skin prick testing

Allergen	Botanical name	Company	Test concentration	Allergen group	Allergen exposure
Alder	*Alnus*	Allergopharma, Reinbek, Germany	50,000 BE/ml	Tree pollen species	Seasonal
Hazel	*Corylus*	Allergopharma, Reinbek, Germany	50,000 BE/ml		
Cypress	*Cupressus*	Stallergenes, Kamp‐Lintfort, Germany	100 IC/ml		
Ash	*Fraxinus*	Leti‐Novartis, Witten, Germany	1%		
Birch	*Betula*	Allergopharma, Reinbek, Germany	50,000 SBE/ml		
Plane tree	*Platanus*	Stallergenes, Kamp‐Lintfort, Germany	100 IC/ml		
Olive	*Olea europaea*	Stallergenes, Kamp‐Lintfort, Germany	100 IR/ml		
Sweet grasses inclusive Timothy grass exclusive Bermuda grass	*Pocaceae* inclusive *Phleum pratense* exclusive *Cynodon dactylon*	ALK‐Abelló, Hamburg, Germany	100 HEP	Grass pollen species	
Wall pellitory	*Parietaria judaica*	ALK‐Abelló, Hamburg, Germany	10 HEP	Weed pollen species	
Mugwort	*Artemisia*	Stallergenes, Kamp‐Lintfort, Germany	100 IR/ml		
Ragweed	*Ambrosia*	ALK‐Abelló, Hamburg, Germany	1:100 g/V		
Dust mite	*Dermatophagoides farinae*	Stallergens, Kamp‐Lintfort, Germany	100 IR/ml	Mite species	Perennial

Abbreviations: BE, biological unit; G/V, weight/volume; HEP, histamine equivalent prick; IC, index of concentration; IR, index of reactivity; SBE, standardized biological unit.

ISAC technology (Fisher Scientific GmbH, Schwerte, Germany; 112 allergen components) was run in the study center in Aachen.^18^, ^19^ IgE data were displayed in ISU, and ISU‐values ≥0.3 were evaluated positive. The pollen allergen components to which sIgE levels were assessed are given in Table 2. The dust mite component Der f 2 was included as perennial control component. In addition, sIgE levels to the pollen pan‐allergen components Bet v 2, Hev b 8, Mer a 1, Phl p 12 (profilins), and Bet v 4 and Phl p 7 (procalcins) were analyzed. Cup a 1, Cry j 1, Pla a 2, Ole e 1, Cyn d 1, Art v 1, Amb a 1, Sal k 1, and Der f 2 were purified native proteins, and Aln g 1, Cor a 1.0101, Bet v 1, Phl p 1, Par j 2, Pla l 1, Che a 1, and the pollen pan‐allergen components were of recombinant origin. Fra e 1, a main allergen component of ash, was not included in the ISAC panel.

**TABLE 2:** Pollen allergen components to which sIgE levels, measured by ISAC technology, were assessed

Allergen component	Allergen source	Details given by the company	Allergen group	Allergen exposure
Aln g 1	Alder	PR‐10 protein	Tree pollen species	Seasonal
Cor a 1.0101	Hazel	PR‐10 protein		
Cup a 1	Cypress	Pectate lyase, mainly species specific		
Cry j 1	Japanese cedar	Pectate lyase, mainly species specific		
Bet v 1	Birch	PR‐10 protein, mainly species specific		
Pla a 2	Plane tree	Polygalacturonase, mainly species specific		
Ole e 1	Olive	Common olive group 5, mainly species specific		
Phl p 1	Timothy grass	Grass group 1, mainly species specific	Grass pollen species	
Cyn d 1	Bermuda grass	Grass group 1, mainly species specific		
Par j 2	Wall pellitory	Lipid transfer protein, mainly species specific	Weed pollen species	
Pla l 1	Plantain	Ole e 1‐related protein, mainly species specific		
Che a 1	Goosefoot	Ole e 1‐related protein, mainly species specific		
Art v 1	Mugwort	Defensin, mainly species specific		
Amb a 1	Ragweed	Pectate lyase, mainly species specific		
Sal k 1	Saltwort	Pectin methylesterase, mainly species specific		
Der f 2	Dust mite	NPC2 family, mainly species specific	Mite species	Perennial

### 2.3 Data on symptom periods and their relation to sensitization data

Information on symptomatic periods was gained from the medical interviews. Patients were asked to highlight all the months over the course of the year with symptoms they would relate to their hay fever/allergic rhinitis disease, hereinafter called “months with symptoms.” The question was applied from the Mini Rhinoconjunctivitis Quality of Life Questionnaire (RQLQ).^20^

To relate specific sensitization to months with symptoms, for each month of the year the number of respective prick test negative patients with symptoms was set 100% and the number of respective prick test positive patients with symptoms was related to it. For a given test allergen and a given month, this would result in values below, around, or above 100% indicating that the proportion of sensitized patients with symptoms was either lower, equivalent or higher than in unsensitized patients. Taken together with respective exposure periods, the course of the year of this relation would give an idea about the “overall” clinical relevance of the respective sensitization.

### 2.4 Pollen data

Pollen data were provided by the German Pollen Information Service Foundation (PID). Data of 12 pollen types, displayed in Table 3, were acquired from the PID reference monitoring stations Mönchengladbach (NRW) and Munich (Bavaria).

**TABLE 3:** Pollen type data included in this study

Pollen type	Allergens of interest belonging to this pollen type
Alder (*Alnus*)	• Alder (*Alnus*)
Hazel (*Corylus*)	• Hazel (*Corylus*)
Cypress family (*Cupressaceae*)	• Cypress (*Cupressus*)• Japanese cedar (*Cryptomeria japonica*)
Ash (*Fraxinus*)	• Ash (*Fraxinus*)
Birch (*Betula*)	• Birch (*Betula*)
Plane tree (*Platanus*)	• Plane tree (*Platanus*)
Sweet grasses (*Poaceae*)	• Sweet grasses inclusive Timothy grass exclusive Bermuda grass (*Poaceae* inclusive *Phleum pratense* exclusive *Cynodon dactylon*)• Bermuda grass (*Cynodon dactylon*)
Nettle family (*Urticaceae*)	• Wall pellitory (*Parietaria judaica*)
Plantain (*Plantago*)	• Plantain (*Plantago*)
Goosefoot family (*Chenopodiaceae*)	• Goosefoot (*Chenopodium*)• Saltwort (*Salsola*)
Mugwort (*Artemisia*)	• Mugwort (*Artemisia*)
Ragweed (*Ambrosia*)	• Ragweed (*Ambrosia*)

*Note*: Botanical names are given in brackets.

Pollen was collected using 7 days volumetric traps (Hirst type) and samples were analyzed according to the national standard DIN EN 16868.^21^ Due to the limitation of light microscopy and/or time‐consuming determination, Japanese cedar, Bermuda grass, and wall pellitory pollen were not differentiated from other cypress, sweet grasses, and nettle pollen types, respectively.

Except for goosefoot family, pollen count data from 2011 to 2013 were analyzed as these years covered the period when the patients were investigated. For goosefoot family pollen, only data from 1999 to 2001 were available. Data were provided as average daily pollen concentration given in pollen/m^3^ air.

Pollen load was expressed by monthly pollen integrals, calculated by summing the average daily pollen concentrations for each month.

If pollen data were missing at one pollen station, the respective days of the other station were coded “missing” as well to allow better comparison of both stations. The resulting magnitudes of days with no pollen data, specified for low, pre/post, and main season of each pollen type, are given in Table S1.

### 2.5 Data management and statistical analysis

In the context of the abovementioned study on climate change and allergy patient data had been entered either manually (anamnestic data from the questionnaires, prick test data from the patient records) or electronically (ISAC data) into a patient database which had been designed using Access 2007 for Windows (Microsoft Corporation, Redmond, USA). For detailed information on data quality management see.^15^

Data analysis was performed using Excel (Excel 2007 for Windows, Microsoft Corporation) and SPSS (IBM SPSS Statistics, Version 26, New York, USA). Correlation of sensitization data was done by categorizing patients into test negative or test positive and calculating respective Spearman’s rank correlation coefficients for all possible data pairs. If a correlation coefficient was >0.7 data of the respective data pair were considered to highly correlate with each other. Bivariate testing for significant group differences was performed with the Chi square test (and Fisher exact test, respectively). Differences with *p*‐values <0.05 were considered as significantly different.

## 3 RESULTS

### 3.1 Sensitization data

**FIGURE 1:**
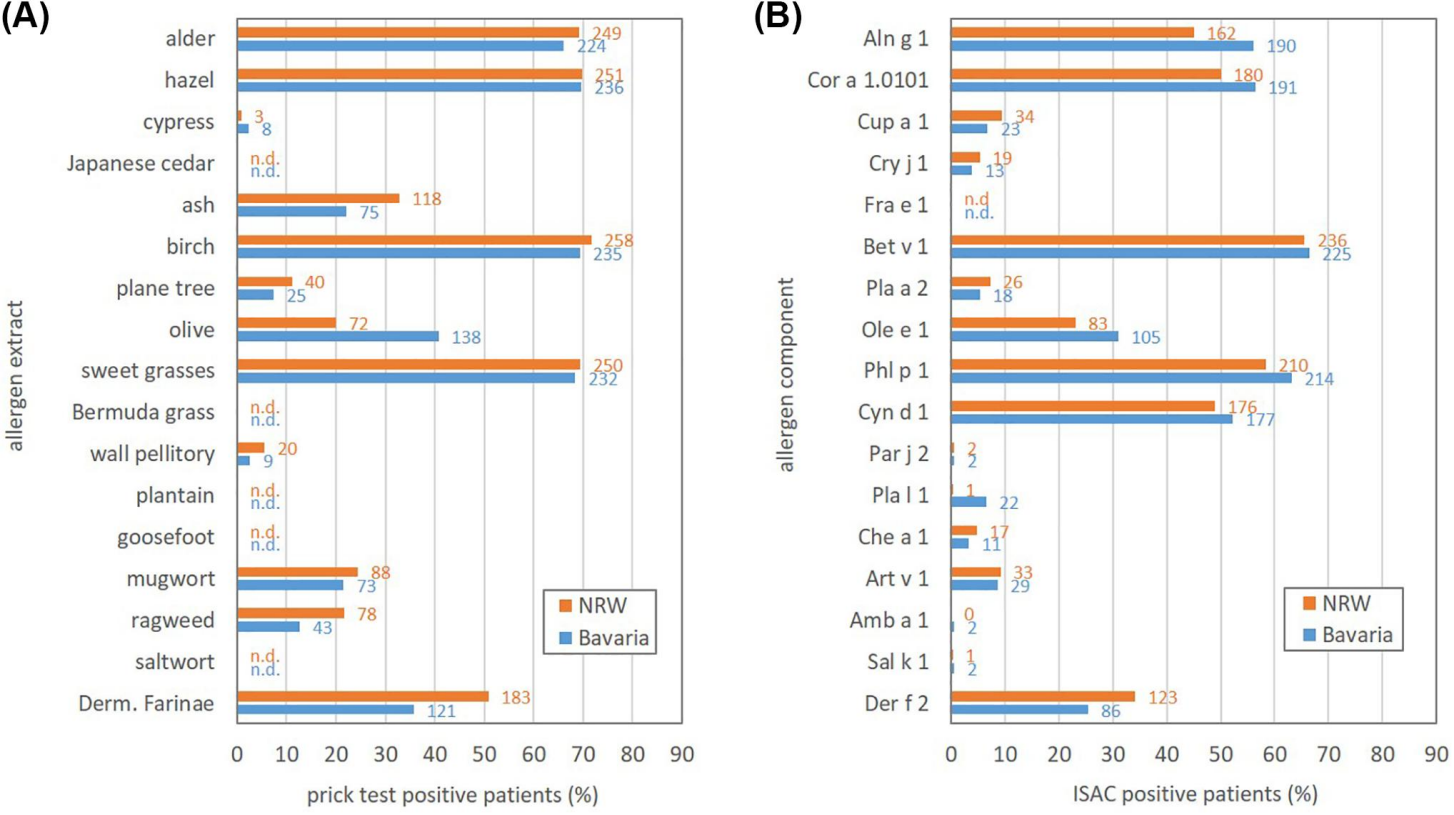
Sensitization to pollen allergens in patients with allergic rhinitis. Sensitization data from 2011 to 2013: (A) prick test data and (B) ISAC data. Allergen extracts respectively allergen components are ordered by the beginning of the flowering season of the respective plant species. Data on sensitization to a mite species, being a perennial respiratory allergen, are given at the end. Values behind bars show respective patient numbers. Orange bars and numbers: NRW. Blue bars and numbers: Bavaria. n.d., no data

Data on sensitization to pollen allergens detected by skin prick testing are given in Figure 1A. Most commonly patients were sensitized to pollen allergens from alder, hazel, birch, and sweet grasses with respective sensitization rates of more than 65% in NRW as well as in Bavaria. With sensitization rates below 65% and above 10%, the list was followed by ash (33%), olive (20%), mugwort (25%), ragweed (22%), and plane tree (11%) in NRW and olive (41%), ash (22%), mugwort (22%), and ragweed (13%) in Bavaria. Sensitization rates below 10% were measured for wall pellitory (6%) and cypress (1%) in NRW and plane tree (7%), wall pellitory (3%) and cypress (2%) in Bavaria.

Data on sensitization to pollen allergens detected by ISAC technology are given in Figure 1B. Most commonly patients were sensitized to components of alder, hazel, birch, and Timothy grass, rates ranging from 45% for Aln g 1 (alder) in NRW to 66% for Bet v 1 (birch) in Bavaria. Cyn d 1 (Bermuda grass) showed sensitization rates of 49% in NRW and 52% in Bavaria. Sensitization against Ole e 1 (olive) was detected in 23% of NRW patients and 31% of Bavarian patients. Sensitization rates between 10% and 1% were detected for Cup a 1 (cypress; 9%), Art v 1 (mugwort; 9%), Pla a 2 (plane tree; 7%), Cry j 1 (Japanese cedar; 5%), and Che a 1 (goosefoot; 5%) in NRW and Art v 1 (mugwort; 9%), Cup a 1 (cypress; 7%), Pla l 1 (plantain; 7%), Pla a 2 (plane tree; 5%), Cry j 1 (Japanese cedar; 4%), and Che a 1 (goosefoot; 3%) in Bavaria. Sensitization rates below 1% were found for Par j 2 (wall pellitory; 0.6%), Pla l 1 (plantain; 0.3%), Sal k 1 (saltwort; 0.3%), and Amb a 1 (ragweed; 0%) in NRW and Par j 2 (wall pellitory; 0.6%), Sal k 1 (saltwort; 0.6%), and Amb a 1 (ragweed; 0.6%) in Bavaria.

With respect to pollen pan‐allergen components, 17% (119/699) of patients (NRW: 16% [56/360], Bavaria: 19% [63/339]; *p* = 0.287) were sIgE positive to at least one of them. Exclusion of these patients from analysis did not significantly change the pollen sensitization pattern (Figure S1).

### 3.2 Correlation of sensitization data

Partially, sensitization data highly correlated which each other, also across both test principles. Data pairs with a correlation coefficient >0.7 are given in Table 4. Correlation coefficients of all data pairs are given in Table S2.

**TABLE 4:** Correlation of sensitization data: data pairs with high correlation

Data pair	Correlation coefficient (*p* value)
Prick test **Alder**/prick test **hazel**	0.901 (0.000)
Prick test **Alder**/prick test **birch**	0.888 (0.000)
Prick test **hazel**/prick test **birch**	0.857 (0.000)
Prick test **Alder**/ISAC test **Bet v 1** (birch)	0.852 (0.000)
Prick test **hazel**/ISAC test **Bet v 1** (birch)	0.846 (0.000)
Prick test **birch**/ISAC test **Bet v 1** (birch)	0.847 (0.000)
Prick test **sweet grasses**/ISAC test **Phl p 1** (Timothy grass)	0.783 (0.000)
Prick test ***Derm. Farinae***/ISAC test **der f 2** (*Derm. farinae*)	0.707 (0.000)
ISAC test **Aln g 1** (Alder)/ISAC test **Cor a 1.0101** (hazel)	0.850 (0.000)
ISAC test **Aln g 1** (Alder)/ISAC test **Bet v 1** (birch)	0.724 (0.000)
ISAC test **Cor a 1.0101** (hazel)/ISAC test **Bet v 1** (birch)	0.764 (0.000)
ISAC test **Phl p 1** (Timothy grass)/ISAC test **Cyn d 1** (Bermuda grass)	0.784 (0.000)

*Note*: According to their test results, patients were categorized test negative or test positive. Correlation of test results was assessed by calculating respective Spearman’s rank correlation coefficients. Data pairs with correlation coefficients >0.7 indicating high correlation are shown. Correlation coefficients of all data pairs are given in Table S2.

### 3.3 Relation between sensitization and months with symptoms

Data on the relation between sensitization and months with symptoms are given in Table 5A,B (prick test data) and Table 5C,D (sIgE data).

**TABLE 5:** Relation between sensitization and months with symptoms

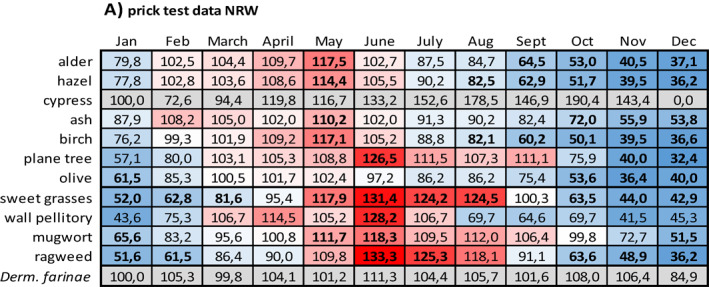 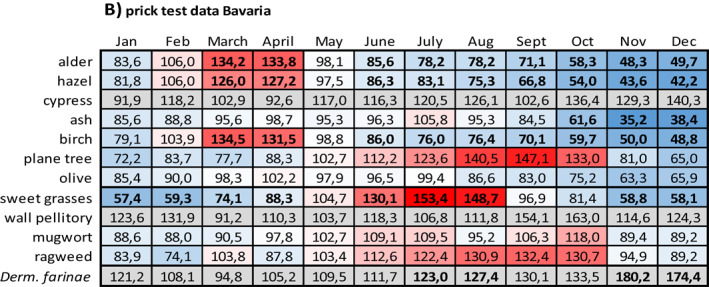 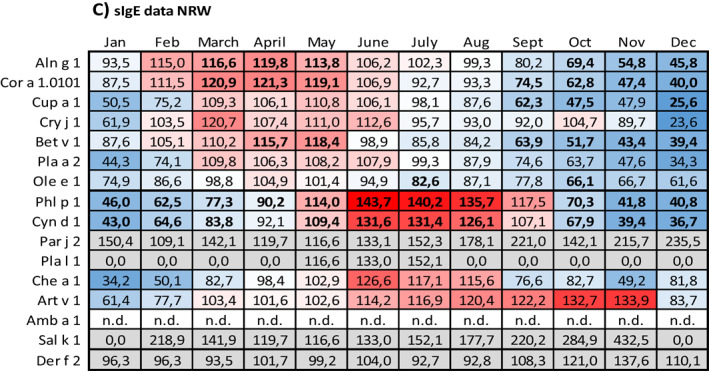 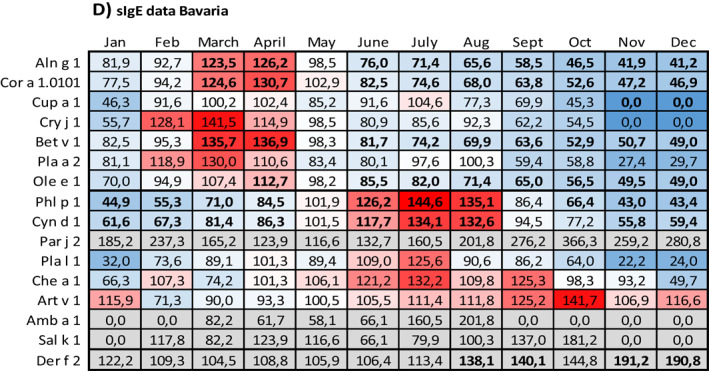

*Note*: For each month of the year, the number of respective prick test negative patients with symptoms was set 100% and the number of respective prick test positive patients with symptoms was related to it. (A) Prick test data from NRW, (B) prick test data from Bavaria, (C) sIgE data from NRW, and (D) sIgE data from Bavaria. The data field colors indicate the position of a given value within the data range: The redder a field, the higher the value in relation to 100%, the bluer, the lower. Proportions based on test positive rates below *n* = 10 and proportions related to dust mite sensitization are highlighted in grey. Proportions based on significant group differences appear in bold. n.d., no data.

Compared to patients without respective pollen sensitization, patients with sensitization to tree pollen more often displayed symptoms in the first months of the year, whereas patients with sensitization to sweet grasses or weed pollen more often displayed symptoms in the mid of the year and in late summer. This pattern especially applied to patients with sIgE diagnosed sensitization. However, it did not apply to all of the tested allergens and especially not to those with sensitization numbers below *n* = 10.

For prick test diagnosed tree pollen sensitization (Table 5A,B), on average, minimum and maximum values were seen in December (NRW: 39.4%; Bavaria: 51.7%; only respective fields with color scaling) and April or May (April: Bavaria, 113.6%; May: NRW, 111.7%; only respective fields with color scaling), respectively. For prick test diagnosed grasses or weed pollen sensitization respective values were seen in December or February (December: NRW, 44.0%; February: Bavaria; 73.8%) and June or July (June: NRW, 127.8%; July: Bavaria, 128.4%), respectively.

For sIgE diagnosed tree pollen sensitization (Table 5C,D), on average, minimum and maximum values were seen in December (NRW: 38.6%; Bavaria: 30.8%; only respective fields with color scaling) and March (NRW: 112.3%; Bavaria: 123.3%; only respective fields with color scaling), respectively. For sIgE diagnosed grasses or weed pollen sensitization, respective values were seen in December or January (December: Bavaria, 58.6%; January: NRW, 46.2%) and June or July (June: NRW, 129.0%; July: Bavaria, 129.6%), respectively.

Compared to patients without sensitization to the perennial allergen dust mite, patients with dust mite sensitization either showed almost no seasonal fluctuations at all (prick test diagnosed patients in NRW, Table 5A) or more often displayed symptoms in the winter season (prick test diagnosed patients in Bavaria, Table 5B; sIgE diagnosed patients in NRW and Bavaria, Table 5C,D).

Respective raw data are given in Table S3 (prick test data) and Table S4 (sIgE data), and all *p* values are given in Table S5.

### 3.4 Pollen data

Pollen data from Mönchengladbach/NRW and Munich/Bavaria from the years 2011 to 2013^2^ were analyzed with respect to monthly pollen integrals. Data aggregated from 2011 to 2013 are shown as respective median values of the monthly pollen integrals (Table 6). Yearly data are given in Table S6.

**TABLE 6:** Monthly pollen integrals from Mönchengladbach/NRW and Munich/Bavaria

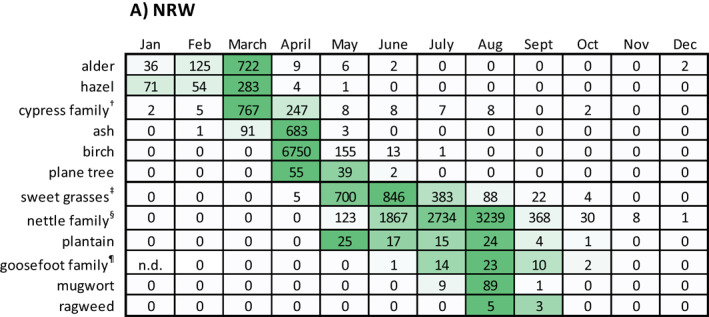 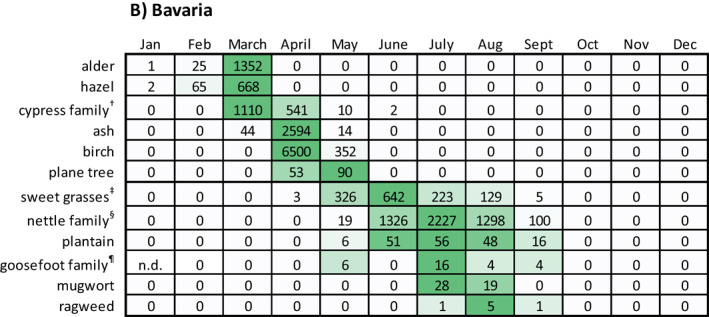

*Note*: Data represent median values of the years 2011–2013 (exception: goosefoot family pollen, 1999–2001): (A) data from Mönchengladbach/NRW and (B) data from Munich/Bavaria. For a given pollen type, the data field color indicates the position of a given value within the data range, the darker a field, the higher the value. n.d., no data.

^†^Could include among others cypress and Japanese cedar pollen.

^‡^Could include among others Timothy grass and Bermuda grass pollen.

^§^Could include among others wall pellitory pollen.

^¶^Could include among others goosefoot and saltwort pollen.

As expected, tree pollen types were mainly detectable in the first months of the year, whereas sweet grasses and weed pollen were mainly detectable in the mid of the year and in late summer.

## 4 DISCUSSION

Here, we show data on sensitization to 16 pollen allergens in patients with physician‐diagnosed allergic rhinitis from two German federal states. We correlate sensitization data with each other and relate them to months with symptoms. Furthermore, we show respective data on regional pollen load.

### 4.1 Sensitization to frequent versus non‐frequent allergens–pharmaceutical and clinical implications

From the 16 allergens analyzed, four are explicitely within the scope of the German TAO and from this point of view can be considered as frequent allergens: alder, hazel, birch and sweet grasses. Population‐based data from DEGS1 showed that these allergens were indeed the most frequent ones with respect to sensitization in German adults.^5^ However, the same study also showed sensitization to other pollen allergens albeit to a lesser extent, and concluding from patient‐based data the GAL^2^EN network has recommended a prick test panel of 10 pollen allergens to be tested in European patients. Apart from pollen of the birch family and sweet grasses this panel included cypress, mugwort, olive *or* ash, plane tree, ragweed and wall pellitory.^5^, ^17^

The prick test data provided by our study confirm the clinical relevance of the GAL^2^EN prick test panel: Each of the test allergens recommended by GAL^2^EN induced positive prick test reactions in at least some of our study patients even if the number greatly varied (range from 3 Cypress‐reactive to 258 Birch‐reactive patients). Additional to the GAL^2^EN prick test panel both olive *and* ash were included in our study. More than 20% of our patients showed positive reactions to olive and ash, respectively, but not all of these positive reactions can be explained by “cross‐reactivity” to ash and olive, respectively, as already described elsewhere.^15^ Currently, in Germany field‐grown olive does not exist and significant olive pollen load has not been found so far. So, olive‐but‐not‐ash reactive patients may have become sensitized to olive on the basis of travelling to respective exposure regions or may cross‐react for instance to other pollen of the family *Oleaceae* like the insect‐pollinated forsythia or Jasmine.^15^ In contrast to olive, ash is endemic in Germany and pollen load is documented. Thus, ash should be included in a German routine prick test panel.

By using ISAC technology we were able to extent our sensitization study to Japanese cedar, Bermuda grass, plantain, goosefoot, and saltwort. Japanese cedar, Bermuda grass and saltwort are not widespread in Germany but plantain and goosefoot are.^22^ For each of these allergens we could see at least one positive reaction in our patient cohort. The number of positive patients ranged from 1 Pla l 1 (plantain)‐positive to 177 Cyn d 1 (Bermuda grass)‐positive patients. The high sensitization rates to Bermuda grass are very likely due to cross reactivity to Timothy grass, which is endemic in Germany.^22^ The correlation data support this hypothesis.

Our sensitization data support the need for approved test and therapy allergens for both the diagnosis and treatment of frequent but also of non‐frequent pollen allergies. To counteract the current decline in the number of approved test allergens in Germany, pharmaceutical companies can now propose a substantial reduction of the fees needed to approve a given test allergen provided it belongs to the group of non‐frequent allergens.^9^, ^23^ Support for the diagnostic and therapeutic needs of patients with non‐frequent pollen allergies may also come from the initiative “National action group for people with rare allergies” (in German “**N**ationales **A**ktionsbündnis für **M**enschen mit **S**eltenen **A**llergien,” NAMSA), founded in 2017 by the Medical Association of German Allergists (in German “**Ä**rzteverband **D**eutscher **A**llergologen,” AeDA e.V.).^24^

### 4.2 Monitoring of pollen load, sensitization, and symptom periods–prerequisite for adequate patient management

Relating our sensitization data to months with symptoms partly revealed a similar seasonal pattern of “positivity” as seen by analyzing the monthly courses of regional pollen counts. At the group level, this indicates an “overall” clinical relevance of the sensitization data. At the individual level, the clinical relevance of sensitization data needs to be assessed in conjunction with symptom periods and pollen data—a diagnostic chain of three links that has to be considered for every patient presenting with seasonal allergic symptoms. If data on symptom periods, pollen load, and sensitization do not give a clear picture of the causative allergen(s), provocation becomes the fourth link of this chain.

Our data strongly underline the importance of a nationwide pollen and sensitization monitoring for adequate management of allergic patients. Ideally, pollen monitoring should provide continuous real‐time pollen data representative for a given region, and these data should be available for free. The status quo of and perspectives for a nationwide pollen monitoring in Germany have been recently summarized by the interdisciplinary working group “National Pollen Monitoring.”^25^ Sensitization monitoring at the population level is carried out systematically in regular intervals by the Robert Koch Institute.^5^ At the patient level systematic monitoring would also be of use but so far is not established in Germany.

## 5 CONCLUSIONS

Sensitization to non‐frequent pollen allergens has to be considered when examining patients with respective seasonal symptoms, and test and therapy allergens for non‐frequent pollen allergies need to be available. Further prerequisites for adequate patient management would be a nationwide pollen monitoring system giving continuous pollen data and a systematic sensitization monitoring at patient level.

## CONFLICT OF INTEREST

The authors declare that they have no competing interests.

## AUTHOR CONTRIBUTIONS

Conny Höflich designed the study, analyzed, and interpreted patient and pollen data and was a major contributor in writing the manuscript. Galina Balakirski cared for study patients, collected patient data, and substantively revised the drafted work. Zuzanna Hajdu cared for study patients and collected patient data. Jens Malte Baron designed the study and supervised the acquisition and interpretation of sIgE data. Katharina Fietkau quantified and interpreted sIgE data. Henning Bier, Hans F. Merk, Ulrich Strassen, and Wolfgang Dott designed the study. Hans‐Guido Mücke, Wolfgang Straff, Gerda Wurpts, and Amir S. Yazdi interpreted data and substantively revised the drafted work. Adam Chaker designed the study, interpreted data, and substantively revised the drafted work. Stefani T.M. Röseler designed the study, cared for study patients, collected patient data, interpreted data, and substantively revised the drafted work. All authors read and approved the final manuscript.

## CONSENT FOR PUBLICATION

Not applicable.

## Supporting information

Supporting Information S1

## ENDNOTES

^1^
For reasons of simplification, throughout this document “pollen” is used as a synonym for “pollen corn.” For adequate application of “pollen” and “pollen corn,” see.^21^

^2^
Exception: goosefoot family pollen 1999–2001; for further details see the Methods section.
