# Supplementary material for: Management of patients with seasonal allergic rhinitis: Diagnostic consideration of sensitization to non‐frequent pollen allergens
Source: Clin Transl Allergy. 2021 Oct 4;11(8):e12058. doi: 10.1002/clt2.12058 (PMC8488942; doi:10.1002/clt2.12058)
Supplement: Supplementary file 1 — Supporting Information S1 [file CLT2-11-e12058-s001.docx]

Supplemental Data, Figure 1. **Sensitization to pollen allergens in patients with allergic rhinitis and negative sIgE to pollen pan-allergen components.** Sensitization data from 2011 to 2013, (A) prick test data, (B) ISAC data. Patients with sIgE to at least one of the pollen pan-allergen components Bet v 2, Hev b 8, Mer a 1, Phl p 12, Bet v 4 or Phl p 7 had been excluded prior to analysis. Allergen extracts respectively allergen components are ordered by the beginning of the flowering season of the respective plant species. Data on sensitization to a mite species, being a perennial respiratory allergen, are given at the end. Values behind bars show respective patient numbers. Orange bars and numbers: NRW. Blue bars and numbers: Bavaria. n.d.: no data

Supplemental Data, Table 1. **Pollen monitoring: Days with no pollen data.** If pollen data were missing at one pollen station, the respective days of the other station were coded “missing” as well to allow better comparison of both stations. The resulting magnitudes of days with no pollen data, specified for low, pre/post and main season of each pollen type, are given as percentage of maximally possible data days within the respective pollen season. (A) Data from 2011 to 2013. (B) Data from 1999 to 2001. Pollen season was defined according to the pollen calendar Germany 2007-2011 provided by the German Pollen Information Service Foundation (exceptions: cypress - www.pollenflug.nord.de, access date 2017-03-27; goosefoot - www.pollenflug.de, access date 2017-03-30).

Supplemental Data, Table 2. **Correlation of sensitization data.** According to their test results patients were categorized test negative or test positive. Correlation of test results was assessed by calculating respective Spearman’s rank correlation coefficients. Correlation coefficients >0.7 indicating high correlation are highlighted in grey. (A) correlation of prick test data, (B) correlation of sIgE data, (C) correlation of prick test and sIgE data.

Supplemental Data, Table 3. **Relation between prick test diagnosed sensitization and months with symptoms: Raw data for Table 5A,B.** (A) NRW, tree pollen, (B) NRW, sweet grasses and weed pollen, (C) NRW, dust mite, (D) Bavaria, tree pollen, (E) Bavaria, sweet grasses and weed pollen, (F) Bavaria, dust mite. Field values show the percentage of respective patients with symptoms in the respective month. Respective n values are given in Figure 1A.

Supplemental Data, Table 4. **Relation between sIgE diagnosed sensitization and months with symptoms: Raw data for Table 5C,D.** (A) NRW, tree pollen, (B) NRW, sweet grasses and weed pollen, (C) NRW, dust mite, (D) Bavaria, tree pollen, (E) Bavaria, sweet grasses and weed pollen, (F) Bavaria, dust mite. Field values show the percentage of respective patients with symptoms in the respective month. Respective n values are given in Figure 1B.

Supplemental Data, Table 5. **Relation between sensitization and months with symptoms: P values to Table 5.** For each month of the year the number of respective test positive patients with symptoms was compared to the number of respective test negative patients with symptoms. Bivariate testing for significant group differences was performed with the Chi Square test (and Fisher's exact test, respectively). Differences with p values <0.05 were considered significant and are highlighted in grey fields. (A) P values to prick test data from NRW, (B) p values to prick test data from Bavaria, (C) p values to sIgE data from NRW, (D) p values to sIgE data from Bavaria. n.d.: no data

Supplemental Data, Table 6. **Yearly pollen data from Mönchengladbach/NRW and Munich/Bavaria.** Monthly pollen integrals of the years 2011 to 2013 (exception: goosefoot family pollen 1999 to 2001). (A-C) Data from Mönchengladbach/NRW, (D-F) data from Munich/Bavaria. For a given pollen type, the data field color indicates the position of a given value within the data range, the darker a field, the higher the value. ^†^could include amongst others cypress and Japanese cedar pollen, ^‡^could include amongst others timothy grass and Bermuda grass pollen, ^§^could include amongst others wall pellitory pollen, ^¶^could include amongst others goosefoot and saltwort pollen, n.d.: no data
